# The ThRombolysis in UnconTrolled Hypertension (TRUTH) protocol: an observational study on treatment strategy of elevated blood pressure in stroke patients eligible for IVT

**DOI:** 10.1186/s12883-015-0493-z

**Published:** 2015-11-23

**Authors:** T. P. Zonneveld, A. Algra, D. W. J. Dippel, L. J. Kappelle, R. J. van Oostenbrugge, Y. B. W. E. M. Roos, M. J. Wermer, H. B. van der Worp, P. J. Nederkoorn, N. D. Kruyt, for the TRUTH investigators

**Affiliations:** Department of Neurology, Academic Medical Center, Amsterdam, The Netherlands; Department of Clinical Epidemiology, Leiden University Medical Center, Leiden, The Netherlands; The Julius Center, University Medical Center Utrecht, Utrecht, The Netherlands; Department of Neurology and Neurosurgery, Brain Center Rudolf Magnus, University Medical Center Utrecht, Utrecht, The Netherlands; Department of Neurology, Erasmus Medical Center, Rotterdam, The Netherlands; Department of Neurology, Maastricht UMC+, Maastricht, The Netherlands; Department of Neurology, Leiden University Medical Center, Leiden, The Netherlands

**Keywords:** Ischemic stroke, Intravenous thrombolysis, Blood pressure treatment

## Abstract

**Background:**

Intravenous thrombolysis (IVT) with (recombinant) tissue plasminogen activator is an effective treatment in acute ischemic stroke. However, IVT is contraindicated when blood pressure is above 185/110 mmHg, because of an increased risk on symptomatic intracranial hemorrhage. In current Dutch clinical practice, two distinct strategies are used in this situation. The active strategy comprises lowering blood pressure with antihypertensive agents below these thresholds to allow start of IVT. In the conservative strategy, IVT is administered only when blood pressure drops spontaneously below protocolled thresholds. A retrospective analysis in two recent stroke trials showed a non-significant signal towards better functional outcome in the active group; robust evidence for either strategy, however, is lacking. We hypothesize that (I) the active strategy leads to a better functional outcome three months after acute ischemic stroke. Secondary hypotheses are that this effect occurs despite (II) increasing the number of symptomatic intracranial hemorrhages, and could be attributable to (III) a higher rate of IVT treatments and (IV) a shorter door-to-needle time.

**Methods and design:**

The TRUTH is a prospective, observational, cluster-based, parallel group follow-up study; in which participating centers continue their current local treatment guidelines. Outcomes of patients admitted to centers with an active will be compared to those admitted to centers with a conservative strategy. The primary outcome is functional outcome on the modified Rankin Scale at three months. Secondary outcomes are symptomatic intracranial hemorrhage, IVT treatment and door-to-needle time. We based our sample size estimate on an ordinal analysis of the mRS with the “proportional odds” model. With the aforementioned signal observed in a recent retrospective study in these patients as an estimate of the effect size and with alpha 0 · 05, this analysis would have an 80 % power with a total number of 600 patients. Corrections for expected imbalance in group size and clustering effects resulted in a sample size of 1235 patients.

**Discussion:**

The TRUTH is the first large prospective study specifically studying IVT-candidates with elevated blood pressure, and has the potential to change clinical practice and optimize acute stroke care in these patients.

## Background

### Elevated pre-treatment blood pressure

Intravenous thrombolysis (IVT) with recombinant tissue plasminogen activator (rtPA) is an effective treatment for acute ischemic stroke [1]. Unfortunately, the number of stroke patients treated with rtPA is still limited [2]. One of the main reasons that patients are withheld IVT is a pre-treatment blood pressure (BP) above systolic BP of 185 mmHg or a diastolic BP > 110 mmHg. These BP thresholds were introduced in the National Institute of Neurological Disorders and Stroke (NINDS) rtPA Stroke Study [3], and were based on pilot studies in which high diastolic BP levels were associated with an increased risk of symptomatic intracerebral hemorrhage (sICH) after treatment with rtPA [4]. In a recent post-hoc analysis in the PRomoting ACute Thrombolysis in Ischemic StrokE (PRACTISE) [5] trial and the Preventive Antibiotics in Stroke Study (PASS) [6], the prevalence of elevated pre-treatment BP in patients otherwise eligible for IVT was substantial: 20 % [7], which is similar to that in a previous retrospective study [8].

### Treatment strategies (active vs. conservative)

In current Dutch clinical practice, and according to national guidelines, neurologists can use two distinct strategies in managing elevated pre-treatment BP in patients otherwise eligible for IVT [9].

The first strategy is active BP lowering with antihypertensive agents, aiming to lower BP below the thresholds to allow IVT. This strategy is in line with recommendations in current international guidelines that BP should be carefully lowered in these patients (Class of evidence: Class IV [10], and Level of Evidence B [11]). However, these recommendations are based on expert opinion only, and several critical remarks can be made regarding this strategy. First, patients with elevated pre-treatment BP were included in the larger IV rtPA trials only if they reached BP thresholds with oral or sublingual antihypertensive medication (e.g. NINDS [3], ATLANTIS [12] and ECASS-III [13]). Therefore, little is known about the efficacy and safety of IVT in patients who require intravenous antihypertensive medication. Secondly, despite lowering BP below IVT thresholds, the risk on sICH could still be increased. Indeed, one retrospective study found an increased relative risk of 2 · 47 (95 % confidence interval, 1 · 15 to 5 · 28) on sICH in patients treated for elevated pre-treatment BP [14]. Two similar studies found similar results but the associations were not statistically significant [15, 16]. Because all three studies were retrospective and relatively small (*n* = 510, 427 and 178 respectively), it is difficult to draw any sound conclusions. Finally, iatrogenic BP lowering in a state of failing cerebral autoregulation could compromise perfusion of the already ischemic penumbra, with subsequent neurological deterioration [17].

The second strategy concerning pre-treatment increased blood pressure is to measure and wait if the blood pressure drops spontaneously. This avoids the possible disadvantages of active BP lowering as mentioned above and is current clinical practice in several Dutch centers, despite international guidelines that advocate an active strategy. In these centers antihypertensive agents are not routinely administered, and IVT is administered only when the BP drops spontaneously below the recommended thresholds. The major disadvantage of this strategy is that fewer patients end up being treated with IVT, when there is no spontaneous decrease in BP [7]. Another disadvantage could be an increase of the door-to-needle time (DNT) while waiting for a spontaneous drop in BP, while an active strategy is associated with only a modest increase in the DNT [18]. However, in a recent study this increase in DNT was not found [7].

### Clinical practice

In contrast to other European countries, Dutch stroke guidelines do not recommend active lowering of blood pressure to date. In order to clarify current clinical practice, and in order to legitimize the proposed trial, we performed an online questionnaire among vascular neurologists in Dutch centers administering IVT. Questionnaires were sent to neurologists in 81 centers and 75 responded (93 %). Of these, 51 (68 %) reported to use an active – and 17 (23 %) a conservative strategy. The remaining 7 centers reported not to have a uniform strategy (unpublished data).

### Aim

The aim of our study is to elucidate whether we should actively lower elevated pre-treatment BP in patients eligible for IVT.

## Methods

### Hypothesis

We hypothesize that in patients with a pre-treatment BP above 185/110 mmHg and otherwise eligible for IVT an active BP lowering strategy leads to [1] a better functional outcome at three months than a conservative watch and wait strategy. Secondary hypotheses are that this effect occurs despite [2] increasing the proportion of symptomatic intracranial hemorrhages, and could be attributable to [3] a higher rate of IVT treatments and [4] a shorter door-to-needle time.

### Design

The ThRombolysis in UnconTrolled Hypertension (TRUTH) is an observational, prospective, multicenter, cluster-based, parallel group follow-up study.

### Patient population

All adult ischemic stroke patients with elevated pre-treatment BP as the only contraindication to delay or withhold treatment with IVT.

### Treatment strategy

Participating centers will continue their current local treatment guideline (either active or conservative strategy). Both the active and conservative strategy have to be formalized in local guidelines, and have to be endorsed by all neurologists executing acute stroke care. These local guidelines do not have to match specific preset criteria, for example regarding route of administration or dosage schemes of antihypertensive agents. Centers are allowed to change their strategy during the course of the study. Patients will receive either strategy, depending on to which center they are admitted.

### Intra-arterial therapy

Treatment with intra-arterial therapy (IAT) is not an exclusion criterion, and in all our analyses we will adjust for this factor. Patients receiving IAT without prior IVT because of elevated BP are also eligible for the TRUTH study as the same contra-indication applies for these patients.

Eleven of the 36 participating centers are currently performing IAT, and ten of these use an active treatment strategy. However, patients enrolled in non-interventional centers eligible for IAT are likely to be referred to an intervention center. Therefore, we expect no major difference in the number of patients receiving IAT between both groups.

### Outcome measures

The primary outcome is the functional outcome on the modified Rankin Scale (mRS) at 90 days assessed with a telephone interview by trained research nurses blinded for the treatment strategy. Secondary outcomes are symptomatic intracranial hemorrhage (sICH), defined as any CT-documented hemorrhage accompanied by clinical deterioration identified as ≥ 4 points increase on the NIHSS compared with the best recorded NIHSS since admission (ECASS II criteria) [19], the rate of patients receiving IVT, and the door-to-needle time recorded as recently defined by Kruyt et al. [20].

### Statistical analysis

The primary analysis will be an ordinal analysis of the seven categories on the mRS, by means of the proportional odds model [21]. In this model, it is assumed that the odds ratio is proportional across all possible dichotomization levels of the ordinal outcome, which will be formally tested with Rao’s score test. To produce an effect estimate (odds ratio with corresponding 95 % confidence interval (CI)) and to allow for covariate adjustment, ordinal logistic regression will be used. Secondly, to assess internal consistence, two fixed dichotomous analyses of the outcome on the mRS will also be performed (good outcome 0–1 vs. poor outcome 2–6, and favorable outcome 0–2 vs. unfavorable 3–6). Secondary outcomes will be analyzed in a Poisson regression analysis (binary outcome; sICH, IVT) or linear regression analysis (continuous outcome; DNT). IVT and sICH rates will be reported as percentages with 95 % CIs, DNTs will be reported as mean with standard deviation or median with interquartile range depending on its distribution.

### Sample size estimates

We based our sample size on the ordinal analysis of the mRS. With the increase in favorable outcome in the active group in the post-hoc analysis as an estimate of the effect size and with alpha 0 · 05, this analysis will have a power of 80 % if we include 600 patients. The distribution between both groups will not be equal; we estimated a ratio of 3:1 in favor of the active strategy based on our aforementioned questionnaire. Since such a distribution leads to a 1 · 33 fold increase of the sample size in a regular chi-square test, we also applied this factor to our sample size estimate, resulting in an estimate of 798 patients.

Because of presumed similarity among patients within preexisting clusters, the variability in response and the power to detect true differences between arms are reduced in a clustered sample. We used the formula by Kerry and Bland to correct for this clustering effect [22]:

$$ \mathrm{D}\mathrm{E}=1+\uprho \left(\left\{\mathrm{c}{\mathrm{v}}^2+1\right\}\mathrm{m}\hbox{-} 1\right) $$

The design effect (DE) is the factor that is ultimately applied to a standard sample size estimate to correct for the clustered design. First, we need to estimate the intracluster coefficient (ρ), which is reflected by the ratio of the variance of a certain variable between and within clusters. With this intracluster coefficient, the mean number of patients per cluster (m), and the coefficient of variation in cluster size (cv, to correct for imbalances in cluster size) [23], the DE is estimated. We used an intracluster coefficient of 0.015 in our calculation. This number was derived from the multicenter cluster-randomized PRACTISE trial [5], in which an ICC of 0.0154 was calculated using their outcome on thrombolysis percentages from 12 Dutch hospitals. We estimated the cv to be 0.50, based on a similar observed coefficient in a study with cluster characteristics comparable to those we expect in our study [24]. Furthermore, we estimate the mean number of patients per cluster to be 30. With these estimations, our design effect is 1.5475 and therefore our effective sample size will be 798 patients with an actual sample size of 1235 patients.

### Feasibility

In the Netherlands, 3760 patients in 86 hospitals were treated with IVT in 2012 [25]. Therefore, we estimate that elevated pre-treatment BP occurs in at least 750 IVT patients per year. In our aforementioned post-hoc analysis, we identified 231 patients with elevated pre-treatment BP of whom 149 received IVT. Therefore, our best estimate of the number of patients in the Netherlands with elevated pre-treatment BP is 1163 per year (231 × 750 / 149).

We estimate the mean number of patients subjected to IVT per participating center per year to be 50. Therefore, in the average center, elevated pre-treatment BP will occur at least 10 times each year. If we include patients for 4 consecutive years, we need 31 centers to participate. As treatment allocation is determined by center, refusal to participate on patients level will be negligible. A list of participating centers and local investigators is supplied in Appendix 1: Executive team.

## Discussion

Elevated pre-treatment blood pressure (BP) is the most common potentially modifiable contraindication for intravenous thrombolysis (IVT). Current international guidelines recommend administering antihypertensive agents in order to lower BP below IVT thresholds. However, this recommendation is based on relatively small, retrospective studies that only report symptomatic intracranial hemorrhage (sICH) as outcome. In contrast to other European countries, Dutch stroke guidelines do not recommend active lowering of blood pressure to date. In most Dutch centers antihypertensive agents are administered (active strategy), whereas in other centers they are not (conservative strategy). In an international Delphi study on contraindications for IVT, no consensus was reached concerning BP management [26]. In our opinion, it remains therefore unclear whether patients with elevated pre-treatment BP should be actively treated with antihypertensive agents or not. The TRUTH study is the first large prospective study specifically designed to elucidate this clinical question.

Following up on the post-hoc analysis in PRACTISE [5] and PASS [6], which revealed a non-significant trend towards better functional outcome in patients subjected to an active treatment strategy, several design options for further study on this clinical question were considered. A randomized design, with randomization either at patient level or at center level, was considered first. However, we deemed this to be unfeasible. Randomization at patient level would be highly susceptible to selection bias, as most treating physicians have a strong preference towards a certain strategy and would therefore be prone to only include specific patients. Besides, in this hyper acute setting where time is of essence, acquiring patient consent for randomization would cost too much time. Randomization at cluster level was deemed unfeasible, as most centers have formulated their treatment strategies in local guidelines and would be unwilling to participate in a trial that would allocate their center randomly to a either strategy.

An observational design is feasible because of the different treatment strategies used in Dutch clinical practice. In some centers, where no uniform strategy was yet defined, one of both strategies was adopted in order to participate in the study. Because of the small number of Dutch centers administering IVT in which no uniform strategy was defined (7 out of 75 in our questionnaire) and the possibility to participate in this study after adopting a uniform strategy, we judge the possibility of selection bias on center level to be very low. The observational design also allowed us to start relatively fast with subject enrollment, an important pragmatic argument in a field moving steadily towards the active strategy despite lack of evidence. Furthermore, we expect that our design results in a much higher participation rate at center level and a higher enrollment rate at patient level in comparison with a randomized design.

As a safety endpoint we used the ECASS-II definition of sICH (any CT-documented hemorrhage accompanied by clinical deterioration identified as ≥ 4 points increase on the NIHSS compared with the best recorded NIHSS since admission). We used this definition because a recent study judged it to be more clinically relevant than most other definitions, in terms of its ability to identify hemorrhages that alter functional outcome three months after acute ischemic stroke [27].

We considered an ordinal analysis of the modified Rankin Scale (mRS) preferable to a binary (dichotomized) analysis for two reasons: First, an ordinal analysis is more efficient in models with a treatment effect present over the entire range of the outcome scale (as we expect in our study); because it retains all information captured by the outcome scale, an ordinal analysis improves study power in such models [28]. Secondly, multiple underlying mechanisms of an active strategy treatment effect might be present simultaneously (higher IVT rate, shorter door-to-needle time, higher sICH rate, iatrogenic penumbral hypoperfusion), and these could express themselves in different directions in different parts of the mRS spectrum. An ordinal analysis is therefore especially appropriate as it captures shifts in both directions (efficacy and safety) in a single analysis [29].

Overall, the TRUTH study is designed to improve clinical practice and optimize acute stroke care in IVT candidates with elevated blood pressure.

### Trial status

Recruitment started in April 2015 and is ongoing (85 participants recruited as of November 1st, 2015).

### Ethics

Written informed consent will be obtained from each patient or legal representative. The patient information describes the purpose and design of the study, and the procedures for recording clinical information and 3-month follow-up. Collection of consent and study enrollment do not have to be done before either treatment strategy is carried out, since the strategy in question is standard of practice in that center. The study protocol, patient information and enrollment procedure were assessed and approved by the Medical Ethics Committee and Review Board of the Academic Medical Center in Amsterdam. Local scientific and ethical committees of each participating center will perform further assessments of local feasibility.

## Appendix 1: TRUTH Investigators

Principal investigators: N.D. Kruyt, MD PhD; P.J. Nederkoorn, MD, PhD.

Steering committee: A. Algra, MD, PhD; D.W.J. Dippel, MD, PhD; L.J. Kappelle, MD, PhD; R.J. van Oostenbrugge, MD, PhD; Y.B.W.E.M. Roos, MD, PhD; M.J. Wermer, MD, PhD; H.B. van der Worp, MD, PhD.

Participating centers (local investigator) as of November 2015: Active: Leiden University Medical Center, Leiden (N.D. Kruyt); Erasmus University Medical Center, Rotterdam (D.W.J. Dippel); University Medical Center Utrecht, Utrecht (H.B. van der Worp); Maastricht University Medical Center Plus, Maastricht (R.J. van Oostenbrugge); Admiral de Ruyter Hospital, Goes (E.W. Peters); Atrium Medical Center, Heerlen (A. Schreuder); Bethesda Hospital, Hoogeveen (J.H. Kwant); Elkerliek Hospital, Helmond (G.S. Grooters); Hospital Gelderse Vallei, Ede (J.M.P. Rovers); Groene Hart Hospital, Gouda (K. de Gans); Maasstad Hospital, Rotterdam (R. Saxena); Maxima Medisch Centrum, Eindhoven (B.C.A.M. van Ginneken); Medical Center Haaglanden Westeinde, The Hague (K. Jellema); Medical Center Leeuwarden, Leeuwarden (W.J. Schuiling); Medisch Spectrum Twente, Enschede (P.J.A.M. Brouwers); Radboud UMC, Nijmegen (E. Richard); Reinier de Graaf Gasthuis, Delft (L.A.M. Aerden); Rijnland Hospital, Leiderdorp (E.L.L.M. de Schryver); Rijnstate, Arnhem (S.E. Vermeer); St. Elisabeth Hospital, Tilburg (J.H. van Tuijl); St. Franciscus Hospital, Rotterdam (F.H. Vermeij); St. Jans Gasthuis, Weert (H. Lövenich); Hospital St. Jansdal, Harderwijk (A. Bijl-van Dijk); Slotervaart Hospital, Amsterdam (F.H.M. Spaander); VieCuri, Venlo (A.M.H.G. van der Heijden); Vlietland Hospital, Schiedam (C.L. Alblas). Conservative: Academic Medical Center Amsterdam, Amsterdam (P.J. Nederkoorn); Albert Schweitzer Hospital, Dordrecht (H. Kerkhoff); Kennemer Gasthuis, Haarlem (F. de Beer); Rode Kruis Hospital, Beverwijk (W.D.M. van der Meulen); VU University Medical Center, Amsterdam (M.C. Visser); Medical Center Alkmaar, Alkmaar (P. Halkes); Onze Lieve Vrouwe Gasthuis, Amsterdam (V.I.H. Kwa); St. Lucas Andreas Hospital, Amsterdam (R.M. van den Berg – Vos); Tergooiziekenhuizen, Hilversum (J.R. de Kruijk); Westfriesgasthuis, Hoorn (T.C. van der Ree).

## Abbreviations

BP: blood pressure
DE: design effect
DNT: door-to-needle time
IAT: intra-arterial therapy
IVT: intravenous thrombolysis
mRS: modified rankin scale
NINDS: National Institute of Neurological Disorders and Stroke
PASS: preventive antibiotics in stroke study
PRACTISE: PRomoting ACute Thrombolysis in Ischemic StrokE
rtPA: recombinant tissue plasminogen activator
sICH: symptomatic intracranial hemorrhage
TRUTH: thrombolysis and uncontrolled hypertension
